# The PlA1/A2 Polymorphism of Glycoprotein IIIa as a Risk Factor for Stroke: A Systematic Review and Meta-Analysis

**DOI:** 10.1371/journal.pone.0100239

**Published:** 2014-07-02

**Authors:** Christopher N. Floyd, Benjamin H. Ellis, Albert Ferro

**Affiliations:** Department of Clinical Pharmacology, Cardiovascular Division, British Heart Foundation Centre of Research Excellence, King's College London, London, United Kingdom; University Heart Center Freiburg, Germany

## Abstract

**Background:**

The PlA1/A2 polymorphism of glycoprotein IIIa (GPIIIa) has been reported to be associated with risk of stroke in some studies, although other studies suggest no such association. This meta-analysis and systematic review was conducted to investigate the hypothesis that carriage of the PlA2 allele is a risk factor for stroke.

**Methods:**

Electronic databases (MEDLINE and EMBASE) were searched for all articles evaluating carriage of the PlA2 allele and the incidence of stroke. Pooled odds ratios (ORs) were calculated using fixed-effect and random-effect models.

**Findings:**

A total of 35 articles were eligible for inclusion, of which 25 studies were suitable for statistical analysis. For carriage of the PlA2 allele, OR 1.12 (*n* = 11,873; 95% CI = 1.03–1.22; p = 0.011) was observed for the incidence of stroke in adults, with subgroup analyses identifying the association driven by stroke of an ischaemic (*n* = 10,494; OR = 1.15, 95% CI = 1.05–1.27; p = 0.003) but not haemorrhagic aetiology (*n* = 2,470; OR = 0.90, 95% CI = 0.71–1.14; p = 0.398). This association with ischaemic stroke was strongest in individuals homozygous for the PlA2 allele compared to those homozygous for wild-type PlA1 (*n* = 5,906; OR = 1.74, 95% CI = 1.34–2.26; p<0.001). Subgroup analysis of ischaemic stroke subtypes revealed an increased association with stroke of cardioembolic (*n* = 1,271; OR 1.56, 95% CI 1.14–2.12; p = 0.005) and large vessel (*n* = 1,394; OR = 1.76, 95% CI 1.34–2.31; p<0.001) aetiology, but not those of small vessel origin (*n* = 1,356; OR = 0.99, 95% CI 0.74–1.33; p = 0.950). Egger's regression test suggested a low probability of publication bias for all analyses (p>0.05).

**Conclusions:**

The totality of published data supports the hypothesis that carriage of the PlA2 polymorphism of GPIIIa is a risk factor for ischaemic strokes, and specifically those of cardioembolic and large vessel origin.

## Introduction

The platelet fibrinogen receptor is integral to primary haemostasis, since it regulates platelet aggregation and the formation of stable thrombus following vascular injury [1], [2]. The mature fibrinogen receptor is formed from the dimerisation of two glycoprotein (GP) subunits, GPIIb and GPIIIa [3], and has a number of stable allelic variants based on single amino acid substitutions [4]. The PlA2 polymorphism of GPIIIa is formed from the substitution of leucine by proline at position 33, and is one of the more studied variants due to reports that it may be associated with cardiovascular disease.

In 1996, carriage of the PlA2 allele of GPIIIa was reported to be associated with myocardial infarction (MI) when compared to subjects homozygous for the PlA1 wild-type [5]. Subsequently, there has been much inter-study disagreement as to the presence of any association, although we have recently performed a meta-analysis which clearly demonstrates a significant association between carriage of the PlA2 allele and MI in younger subjects (≤45 years old: *n* = 9,547; odds ratio (OR) 1.205, 95% confidence interval (CI) 1.067–1.360; p = 0.003) [6].

MI and ischaemic stroke are modulated by the same cardiovascular risk factors, although differing arterial specificity for these risk factors underlies subtle differences in their respective pathophysiology [7]. Divergence in aetiology of these pathologies is further illustrated by the observation that conventional cardiovascular risk factors only account for a small proportion of all stroke risk, and familial studies suggest that genetic factors may be more important [8]. Indeed, the major common loci associated with MI have been found to not give a risk profile of similar magnitude for ischaemic stroke, although it has been suggested that they may play a role restricted to specific stroke subtypes [9].

The PlA2 polymorphisms of GPIIIa may transcend these pathophysiological discrepancies as, lying adjacent to the ligand binding site of the fibrinogen receptor, it is positioned to modulate the final common pathway of platelet-mediated thrombosis. Indeed, a meta-analysis published in 2010 considering 187 candidate genes for ischaemic stroke identified 14 studies examining the PlA2 polymorphism, and suggested the presence of a possible association [10], but with no subgroup analyses performed.

Here we present the most comprehensive meta-analysis to date of the association between carriage of the PlA2 allele and ischaemic stroke, with inclusion of 25 studies and 11,873 subjects. Subgroup analyses based on subject demographics and ischaemic stroke subtypes are presented. Additionally, we report the first meta-analysis examining the possible association of the PlA1/A2 polymorphism with haemorrhagic stroke in 1,621 subjects, to further explore the relationship between this polymorphism and cerebrovascular events.

## Methods

### Search strategy

Electronic databases (MEDLINE and EMBASE) were searched up until 30^th^ July 2013 for all articles evaluating carriage of the PlA2 polymorphism of GPIIIa and the incidence of stroke. The medical subject headings (MeSH) terms used in the primary literature search were ‘stroke’, ‘cerebrovascular’, ‘subarachnoid’, ‘subdural’, and ‘extradural’ in combination with ‘glycoprotein IIIa’, ‘integrin beta3’ and their associated synonyms. No language restrictions were in place during the primary search. A secondary search of the literature was performed by hand, based on all potentially relevant articles identified from the primary search.

### Inclusion criteria and data extraction

Articles were considered suitable for inclusion in the systematic review if they studied association between carriage of the PlA2 polymorphism and risk of stroke. Studies performed wholly in children (<18 years) were excluded, although studies that considered ‘young adults’ (often defined as 15–44 years) were included. Non-English language articles were included if sufficient information could be extracted from the abstract.

In the case of studies suitable for statistical analysis, the following data were extracted where available: the number of cases/controls for each genotype (or calculated OR where raw data were not available), stroke subtype and population demographics (age, sex, ethnicity and the presence of traditional cardiovascular risk factors). For duplicated studies, the most complete dataset was utilised. Where more than one control group was available, those with cardiovascular risk factors were selected in preference to healthy subjects. Where genotype information was reported for >1 subpopulation as defined by geographic region or ethnic origin, each subpopulation was considered separately in the analyses [11].

### Statistical analysis

Data were analysed using Comprehensive Meta-analysis version 2 software (Biostat Inc., NJ, USA). Pooled ORs were calculated using fixed- and random-effects models, along with the 95% CIs to measure the strength of association. Fixed-effects summary ORs were calculated using the Mantel-Haenszel method [12], and the DerSimonian method was used to calculate random-effects summary ORs [13]. For data where more than one outcome was reported, combined effects were calculated as necessary [14]. Pooled ORs presented in the results were calculated using the fixed-effects model unless otherwise stated.

Tests for heterogeneity were performed for each analysis, with significance set at p<0.05 [15]. I^2^ was also calculated for each analysis, where ≥50% may represent substantial heterogeneity [16]. For assessment of publication bias, we utilised a funnel plot and Egger's regression asymmetry test [17]. In addition, the effect of individual studies on the summary OR was evaluated by re-estimating and plotting the summary OR in the absence of each study.

## Results

The primary search yielded 2,644 unique articles, of which 43 were identified as being potentially relevant following review of title and abstract [18]–. Eleven studies were subsequently discarded following full text review for the following reasons: non-English language (*n* = 1) [20]; no appropriate control group identified (*n* = 1) [21]; duplication of an included dataset (*n* = 4) [22]–[25]; no data presented on the PlA2 polymorphism (*n* = 2) [26], [28]; the PlA2 allele not detected within the subject population (*n* = 4) [58]–[61].

**Figure 1:**
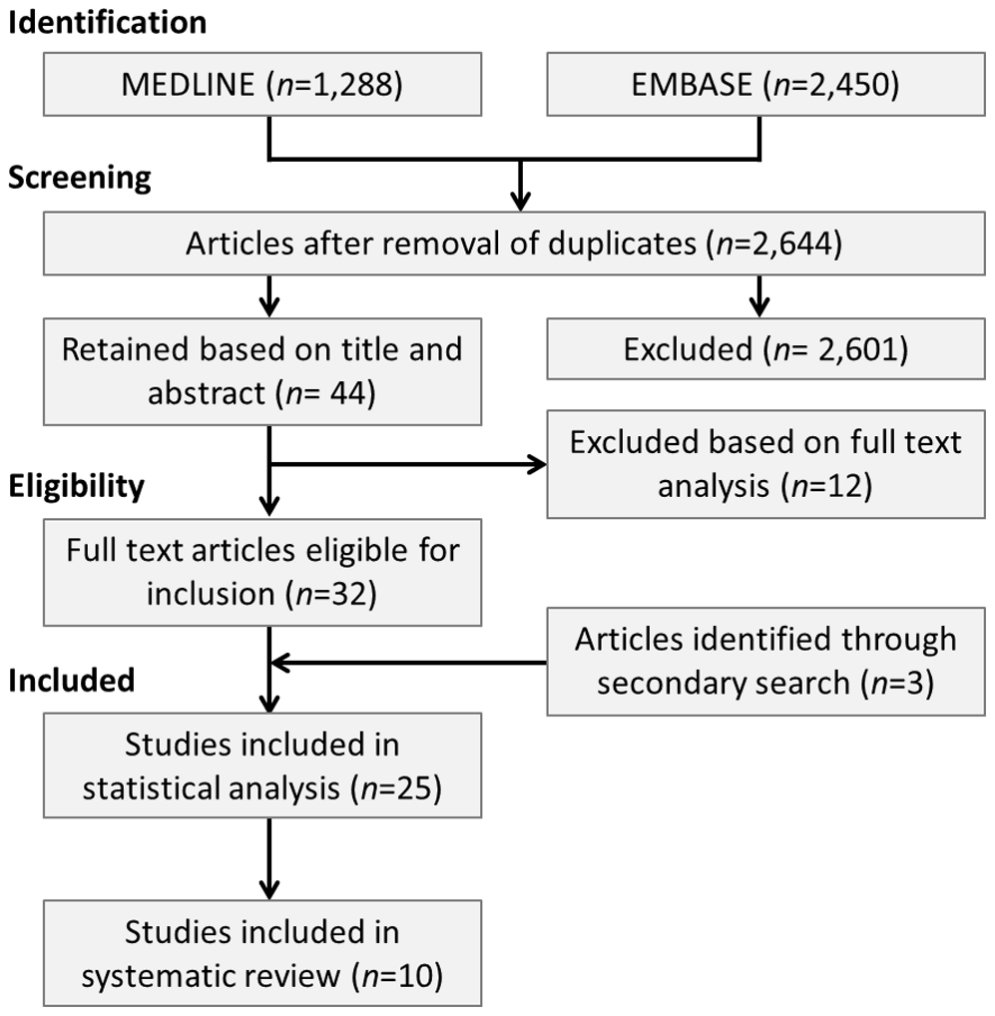
Summary of Search Strategy.

The secondary search yielded an additional three articles [62]–[64] that were suitable for inclusion, resulting in a total of 35 unique articles that met the inclusion criteria (Figure 1). Twenty-five studies contained sufficient data to be included in the meta-analysis [18], [19], [27], [29]–[48], [62], [63] and an additional ten studies were included in the systematic review [49]–[57] [64].

Carriage of the PlA2 allele was significantly associated with total stroke, with an OR 1.12 (*n* = 11,873; 95% CI = 1.03–1.22; p = 0.011). Significant heterogeneity was observed (I^2^ = 56.2%; p<0.001), and the significance of association with the PlA2 allele was lost with application of the random-effects model (OR 1.13; 95% CI = 0.98–1.30; p = 0.087).

**Figure 2:**
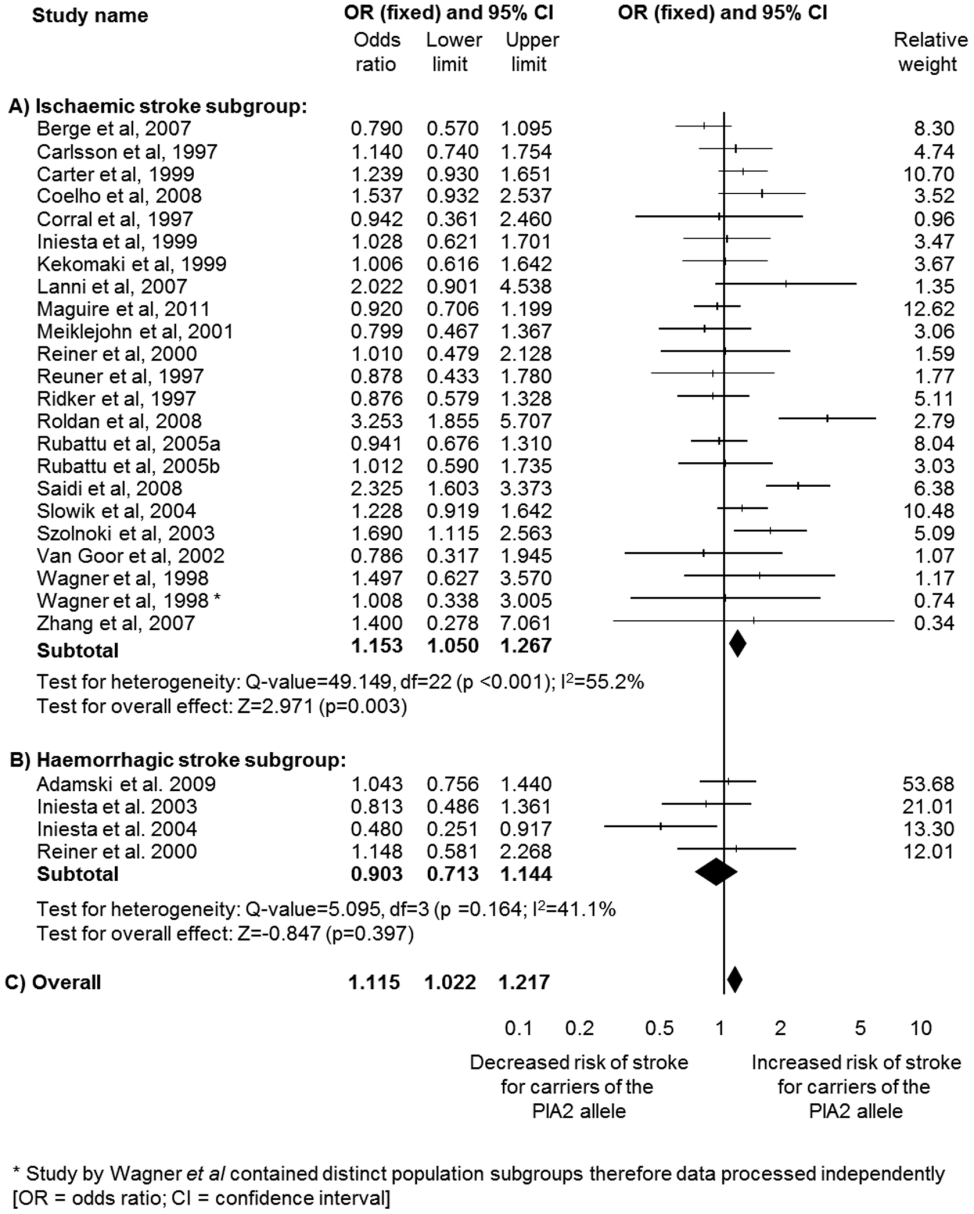
Sub-analysis by stroke type: forest plots for the association of carriage of the PlA2 polymorphism and risk of (A) ischaemic stroke, (B) haemorrhagic stroke and (C) combined haemorrhagic and ischaemic stroke.

Ischaemic stroke was significantly associated with carriage of the PlA2 allele (*n* = 10,494; OR 1.15, 95% CI 1.05–1.27; p = 0.003) (Figure 2A), with significant heterogeneity observed (I^2^ = 55.2%; p<0.001). Analysis using the random-effect model revealed a significant association of similar magnitude (OR = 1.18; 95% CI = 1.01–1.37; p = 0.037). Subgroup analysis of subjects homozygous for the PlA2 polymorphism demonstrated a greatly increased degree of association (*n* = 5,906; OR 1.74, 95% CI 1.34–2.26; p<0.001) (Table 1).

**Table 1:** Level of association between carriage of the PlA2 allele and the risk of stroke.

Analysis	Number of studies	Number of cases/controls	Pooled OR* (95% CI)	Association (p value)	I^2^ (%)
All stroke	25	5,195/6,678	1.12	0.011	56.2
	[18], [19], [27], [29]–[48], [62], [63]		(1.03–1.22)		
Ischaemic stroke	22	4,517/5,977	1.15	0.003	55.2
	[18], [19], [27], [30]–[34], [37]–[48], [63]		(1.05–1.27)		
Haemorrhagic stroke	4	574/1,047	0.90	0.398	41.1
	[29], [35], [36], [40]		(0.71–1.14)		
**Subgroup analyses based on genotype:**					
PlA1/A2 genotype in ischaemic stroke	19	3,948/4,723	1.17	0.003	49.3
	[18], [27], [30]–[34], [38]–[48], [62]		(1.05–1.30)		
PlA2/A2 genotype in ischaemic stroke	18	2,666/3,240	1.74	<0.001	70.0
	[18], [27], [30]–[34], [38]–[47], [62]		(1.34–2.26)		
PlA2/A2 genotype in haemorrhagic stroke	4	426/748	1.11	0.816	0.0
	[29], [35], [36], [40]		(0.47–2.58)		

*OR (odds ratio) calculated using fixed-effects model for carriage of the PlA2 allele versus PlA1 homozygous subjects.

[CI = confidence interval].

The ten studies assessing carriage of the PlA2 allele did not include suitable data for statistical analysis and are summarised in Table 2 [49]–[57] [64]. Three of these studies suggested that carriage of the PlA2 allele increased the risk of ischaemic stroke [49], [51], [52], a single study suggested that PlA2 carriage may be protective [57], and the remaining six study suggested no relationship [50], [53]–[56], [64].

**Table 2:** Summary of studies investigating the association between carriage of the PlA2 polymorphism and ischaemic stroke that were unsuitable for inclusion in the statistical analysis.

Study	Subject characteristics	Comment
Addad *et al*, 2010 [57]	Stable coronary artery disease (n = 188)	Composite endpoint of major adverse cardiovascular events at 1 year was more frequent in subjects homozygous for PlA1 allele
Castro *et al*, 2004 [53]	Homozygous for sickle cell anaemia (n = 97)	No significant association between carriage of the PlA2 allele and risk of occlusive vascular events
Galasso *et al*, 2010 [49]	Hypertensive patients with prior cerebrovascular event (74 cases, 100 controls)	Carriage of the PlA2 allele associated with an increased risk stroke, both in terms of healthy controls and compared to risk of a transient ischaemic attack
Komarov *et al*, 2009 [54]	Stable coronary artery disease (n = 287)	Risk of composite cardiovascular end point was not elevated in patients carrying the PlA2 allele
Lalouschek *et al*, 2007 [64]	Cerebrovascular event in patients <60 years old (450 cases, 817 controls)	No significant association between carriage of the PlA2 allele and risk of stroke or transient ischaemic attack
Mustaffa *et al*, 2009 [50]	Malay ischaemic stroke patients (91 cases, 104 controls)	No difference in allele frequency between stroke patients and healthy blood donors
Pongracz *et al*, 2001 [52]	Hungarian stroke patients (234 cases, 173 controls)	Non-significant increase in carriage of the PlA2 allele among stroke patients >50 years old
Streifler *et al*, 2001 [51]	Carotid artery stenosis (n = 153)	Carriage of the PlA2 allele increased risk of stroke or transient ischaemic attack
Yeh *et al*, 2004 [55]	Stroke patients <50 years old (n = 231)	Carriage of the PlA2 allele was not associated with an increased risk of the composite cardiovascular end point at 1 year
Wei *et al*, 2009 [56]	Ischaemic stroke patients (265 cases, 280 controls)	Distribution of PlA2 allele was not different between ischaemic stroke group or control group

In the case of haemorrhagic stroke, no significant association was observed with either all carriers of the PlA2 allele (*n* = 1,621; OR = 0.90; 95% CI 0.71–1.14; p = 0.398) (Figure 2B) or those homozygous for the PlA2 allele (*n* = 1,174; OR 1.11, 95% CI 0.47–2.58; p = 0.816) (Table 1).

### Subgroup analysis based on aetiology of ischaemic stroke

**Figure 3:**
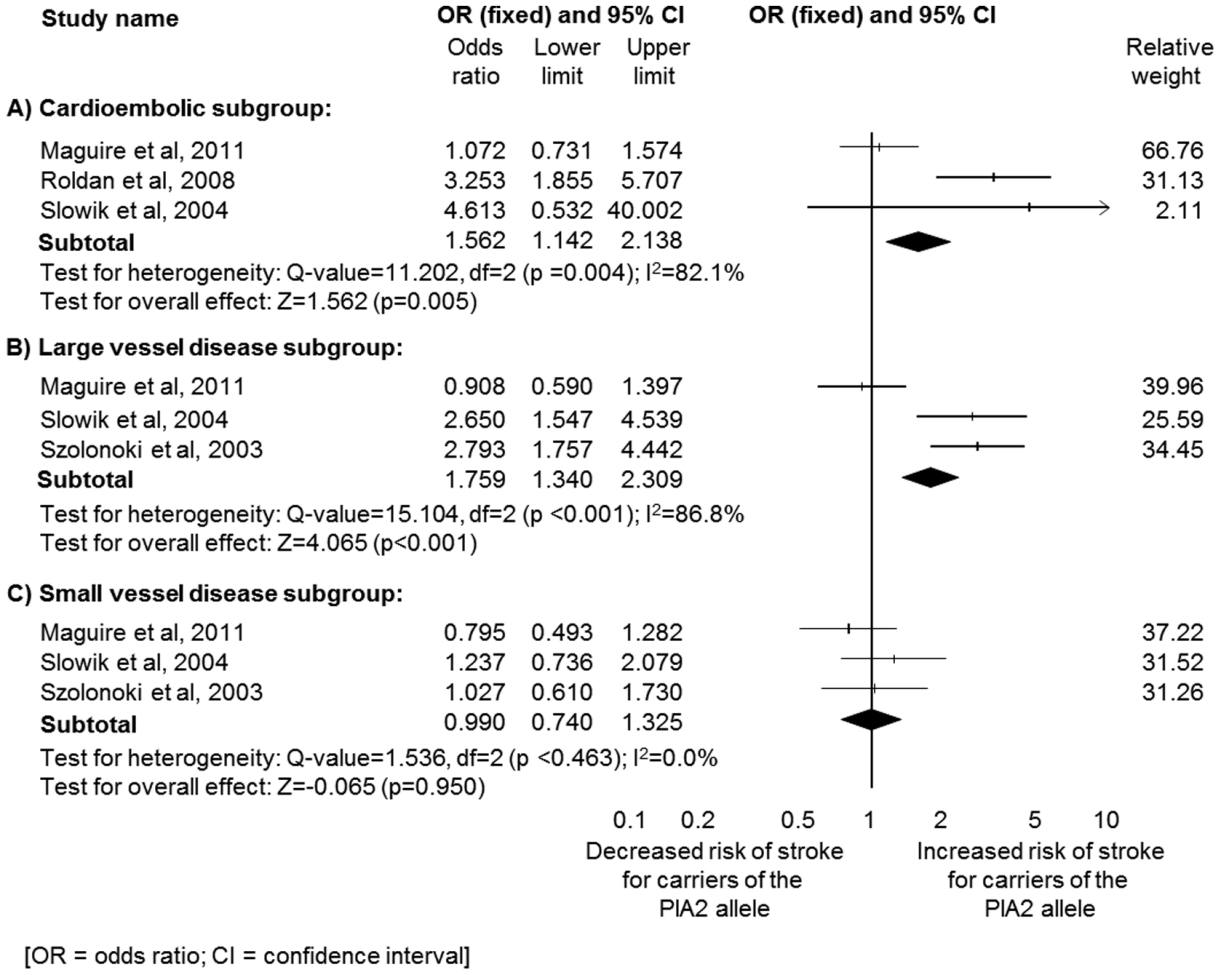
Subgroup analysis based on ischaemic stroke subtype. (A) cardioembolic aetiology, (B) large vessel disease and (C) small vessel disease.

The magnitude of the association observed between carriage of the PlA2 allele and ischaemic stroke was not independent of aetiology of the vascular event (Figure 3). Significant associations were observed with ischaemic stroke secondary to large vessel disease (LVD) (*n* = 1,394; OR 2.76. 95% CI 1.34–2.31; p<0.001) and cardioembolic (CE) disease (*n* = 1,271; OR 1.56, 95% CI 1.14–2.12; p = 0.005), but not with stroke secondary to small vessel disease (SVD) (*n* = 1,356; OR 0.99, 95% CI 0.74–1.33; p = 0.950) (Table 3). Substantial heterogeneity was observed for both the LVD (I^2^ = 86.7%, p<0.001) and CE subgroups (I^2^ = 82.1%, p<0.001).

**Table 3:** Subgroup analyses of the carriage of the PlA2 polymorphism and ischaemic stroke.

Analysis	Number of studies	Number of cases/controls	Pooled OR* (95% CI)	Association (p value)	I^2^ (%)
**Subgroup analysis based on stroke aetiology:**					
Large vessel disease	3	452/942	1.76	<0.001	86.8
	[18], [27], [45]		(1.34–2.31)		
Small vessel disease	3	392/964	0.99	0.950	0.0
	[18], [27], [45]		(0.74–1.33)		
Cardioembolic	3	420/851	1.56	0.005	82.1
	[27], [41], [45]		(1.14–2.12)		
**Subgroup analysis based on subject demographics:**					
Young adult	6	411/1,021	1.27	0.076	0.0
	[31], [33], [40], [42], [46], [47]		(0.98–1.67)		
Caucasian	6	1,832/2,011	1.22	0.009	0.0
	[18], [29], [31], [40], [45], [47]		(1.05–1.42)		
Female	3	172/526	1.28	0.217	0.0
	[40], [45], [47]		(0.87–1.88)		
**Subgroup analysis based on study characteristics:**					
<250 cases	15	1,484/3,012	1.15	0.078	33.1
	[19], [32]–[34], [37]–[42], [46]–[48], [63]		(0.99–1.33)		
≥250 cases	7	2,832/2,965	1.16	0.017	77.6
	[18], [27], [30], [31], [43]–[45]		(1.03–1.31)		

*OR (odds ratio) calculated using fixed-effects model for carriage of the PlA2 allele versus PlA1 homozygous subjects.

### Ischaemic stroke subgroup analyses based on subject demographics

Data were available to calculate pooled ORs based on subject age, ethnicity and sex (Table 3). No subgroup analyses based on the presence or absence of traditional cardiovascular risk factors were possible. Of these subgroup analyses, only Caucasian ethnicity was found to result in a significant association between carriage of the PlA2 allele and ischaemic stroke (*n* = 3,843; OR 1.22, 95% CI 1.05–1.42; p = 0.009).

### Publication bias

Publication bias was assessed by construction of funnel plots and calculation of Egger's regression intercept. All analyses demonstrated a low probability of publication bias (p<0.05). Furthermore, subgroup analyses based on study size did not suggest increased publication of positive results by smaller studies. For studies with <250 cases, OR was 1.15 (n = 4,496; 95% CI 0.99–1.33; p = 0.078), and for studies with ≥250 cases, OR was 1.16 (n = 5,797; 95% CI 1.03–1.31; p = 0.017) (Table 3).

## Discussion

The results of this study demonstrate that carriage of the PlA2 allele of GPIIIa is a strong risk factor for ischaemic stroke. The validity of this association is strengthened by the finding that subjects homozygous for the mutant allele have almost five times the risk of stroke compared to those subjects who are heterozygous.

An age-skewed risk profile caused by a relative decrease in the influence of genetic factors with increasing age might have been expected, given the results of our recent meta-analysis of the association between PlA2 carriage and risk of MI, which demonstrated a stronger association in younger patients [6], [65]. However the association in young adults was found to be of similar magnitude to that in the primary analysis of ischaemic stroke, notwithstanding the non-significant CI. The apparent absence of an age-dependent risk profile may be partially attributed to an underpowered subgroup (441 young adult cases) but also to the fact that unlike in MI, conventional risk factors only account for a small proportion of all stroke risk and that genetic factors are thought to be more important [8]. The association between stroke and carriage of the mutant allele is therefore not obscured in older individuals in the same way as in the MI population.

Subgroup analyses were performed for ethnicity, sex and study size, the results for sex and study size being consistent with the primary analysis suggesting that these factors are not major modulators of the increased risk associated with the polymorphism. Caucasian ethnicity produced a significantly higher association, suggesting that there may be ethnic differences in the impact of carriage of the PlA2 allele. Analysis based on ethnicity was clearly limited by the underlying low prevalence of the PlA2 allele in non-Caucasian populations. The prevalence of the allele in Asians is low, and this was emphasised in three studies of Chinese populations that identified no carriers of the mutant allele across their 554 cases [59]–[61]. Data were not available to perform either quantitative or qualitative analyses based on the presence or absence of conventional cardiovascular risk factors.

### Ischaemic stroke subtypes

Subgroup analyses based on ischaemic stroke subtype identified an increased association between carriage of the PlA2 allele and stroke of CE or LVD aetiology. These findings are based on three studies per subtype and contain low numbers of cases (520 and 452 respectively). However, much published data suggest that platelet activation modulates CE and LVD stroke rather than SVD. In a prospective study of 54 cases of non-CE stroke, those with LVD were found to have significantly higher platelet expression of CD63 during the acute phase, and significantly higher CD62P (P-selectin) expression up to 30 days post event, thus indicating higher levels of platelet activation [66]. The capacity of antiplatelet agents taken within 7 days of an ischaemic stroke to modulate stroke severity has been found to be much greater in LVD than in other stroke subtypes, in a study of 1,622 patients [67]. Moreover, despite the general assumption that CE thrombi are generally platelet-poor, autopsy data from 17 patients who died within 30 days of an ischaemic stroke found that, in CE thrombi, there was a greater proportion of red blood cells and a reduced proportion of fibrin, but no significant difference in the proportion of platelets, when compared to LV thrombi [68].

A further divergence in the aetiology of ischaemic stroke between LVD and SVD may arise because of differences in wall shear stress in the respective vessels. The mechanism by which platelets aggregate is dependent on shear stress, with transient aggregation occurring in the absence of activation at high shear rates [1]. Interestingly, *in vitro* investigations into the effect of the proline substitution on ligand binding have observed increased adhesion to fibrinogen and von Willebrand factor (vWF) in cell culture under conditions of shear stress [69], compared to static systems where no significant differences in maximal binding (B_max_) or dissociation constant (K_d_) were observed [70], [71]. It was previously thought that shear stress was relatively constant throughout the vascular system, but more recent publications suggest that there is significant variability and that the common carotid artery is the site with highest shear [72]. Such differences in shear stress between large and small vessels, with resultant divergent effects on platelet activation, may contribute to the increased association of stroke observed, in carriers of the PlA2 allele, in CE and LVD when compared to SVD.

### Haemorrhagic stroke subgroup

There is a suggestion, from the results of this study, of an apparent decrease in the risk of haemorrhagic stroke in subjects carrying the PlA2 allele. This however was non-significant, but it should be noted that this analysis was based on a relatively small pool of 2,470 subjects, and merits further investigation in future studies. If confirmed, this would give further insight into the mechanism by which the polymorphism affects platelet function. As discussed above, the amino acid substitution lies adjacent to the ligand binding site of the fibrinogen receptor and so may modulate normal platelet function; however, whether this is truly so remains contentious. *Ex vivo* studies have provided conflicting data on the effect that carriage of the PlA2 allele has on platelet fibrinogen binding and on platelet aggregation, in both healthy and ischaemic stroke cohorts [70], [73], [74] [75]. The PlA1/A2 polymorphism has also been hypothesised to modulate efficacy of anti-platelet drug therapy, and hence to influence cardiovascular risk whilst on such therapy; however, a recent meta-analysis suggests that this is unlikely to be the case [76].

Observations from *in vivo* studies appear to be more consistent, with carriage of the PlA2 allele resulting in a reduction in bleeding time both pre- and post-aspirin therapy in healthy subjects [77], [78]. Additionally, carriage of the mutant allele was found to reduce peri-operative bleeding following coronary artery bypass grafting in aspirin-naïve subjects [79].

The totality of these experimental findings, when viewed in the context of the clinical data reported in the present study, suggests that carriage of the PlA2 allele does indeed result in modulation of platelet function leading to increased activation and aggregation. The clinical manifestation of this modulation is an increased propensity for thrombus formation. The discrepancy with *ex vivo* findings may possibly be due to the impact of the polymorphism being dependent on interaction of platelets with the vasculature, and this hypothesis warrants further investigation in future *in* vitro studies.

### Study limitations

There are a number of limitations to the study presented here that are inherent to the data available. Due to the increase in the prevalence of traditional cardiovascular risk factors with age, it is preferable to include data that are adjusted for these factors as the contribution of a single gene polymorphism to a multifactorial, polygenic process is likely to be subtle [11] [65]. Data were not available to perform such analysis nor for subgroup analyses based on non-Caucasian ethnicity or female sex, and the data for young adults were limited.

The majority of strokes occur out-of-hospital with many patients not surviving the index event [80]. Inclusion into the studies reported here necessitated survival for a period of time post-event, and so the presence of a mortality bias may attenuate the true association. There is no post-mortem study equivalent for the Helsinki Sudden Death Study for stroke [81] , but a polymorphism in GPIIb has been shown to increase the risk of fatal stroke and to decrease post-stroke survival suggesting the possibility of similar findings with carriage of the PlA2 allele [82].

## Conclusions

We have demonstrated that carriage of the PlA2 polymorphism of GPIIIa is associated with an increased risk of ischaemic stroke in adults, and this risk is higher in homozygous subjects and those with strokes of CE and LVD aetiology. This risk is likely to be modulated by increased platelet activity that, in conjunction with interaction with the vascular wall, results in increased thrombus formation. The magnitude of the increased risk in PlA2 carriers is similar to that seen in MI, and suggests that PlA1/A2 genotyping may add usefully to risk stratification for patients at risk of stroke.

## Supporting Information

Checklist S1 **PRISMA Checklist.**
(DOC)
